# Accumulation Mechanisms of CD4^+^CD25^+^FOXP3^+^ Regulatory T Cells in EBV-associated Gastric Carcinoma

**DOI:** 10.1038/srep18057

**Published:** 2015-12-17

**Authors:** Na-na Zhang, Jian-ning Chen, Lin Xiao, Fang Tang, Zhi-gang Zhang, Yi-wang Zhang, Zhi-ying Feng, Ye Jiang, Chun-kui Shao

**Affiliations:** 1 Department of Pathology, The Third Affiliated Hospital, Sun Yat-sen University, No. 600 Tianhe Road, Guangzhou 510630, China

## Abstract

Approximately 10% of gastric carcinomas are associated with Epstein-Barr virus (EBV) and are defined as EBV-associated gastric carcinomas (EBVaGCs). EBVaGCs are known to be accompanied by massive CD8^+^ cytotoxic T cell (CTL) infiltration; however, adoptive cellular immunotherapy based on EBV-specific CD8^+^ CTLs has been explored with limited success. Because regulatory T cells (Tregs) are regarded as a critical hurdle in anti-tumour immunity, we assessed the distribution of Tregs in 45 cases of EBVaGC and 45 cases of EBV-negative gastric carcinoma (EBVnGC) with matched clinicopathological parameters by immunohistochemistry. We showed that Tregs were significantly increased in EBVaGC compared to EBVnGC (15.92 ± 11.45/HPF vs. 8.45 ± 6.16/HPF, *p* = 0.001). In addition, we explored the accumulation mechanisms of Tregs in EBVaGC by using EBV (+) gastric carcinoma cell lines SNU719 and GT39 as *ex vivo* models. When peripheral blood mononuclear cells (PBMCs) were co-cultured with EBV (+) gastric carcinoma cell lines, the Treg frequency increased, and they underwent phenotypic and functional changes. The enhanced recruitment by CCL22 produced by EBVaGC cells, the decreased emigration due to CCR7 downregulation on the Treg surface, the higher proliferation rate, and the lower apoptosis rate of Tregs at tumour sites may promote the accumulation of Tregs in EBVaGC.

Epstein-Barr virus (EBV) is an oncogenic virus that is closely associated with a wide range of human lymphoid and epithelial malignancies, including Burkitt lymphoma (BL), Hodgkin lymphoma (HL), nasal NK/T cell lymphoma, nasopharyngeal carcinoma (NPC) and a subset of gastric carcinoma defined as EBV-associated gastric carcinoma (EBVaGC)^1^.

EBVaGC is defined by the presence of EBV in gastric carcinoma cells, as demonstrated by EBV-encoded RNA (EBER) *in situ* hybridization. EBVaGC accounts for approximately 10% of gastric carcinoma worldwide^2^. It shows some distinct clinicopathological characteristics, such as male predominance, predisposition to the proximal stomach, and a high proportion in diffuse-type gastric carcinomas^2^. Moreover, EBVaGCs are usually accompanied by massive lymphocyte infiltration^2^. These infiltrating lymphocytes are predominantly CD8^+^ T cells with high proliferative capacity and cytotoxicity, many of which express perforin and granzyme B^3^,^4^. *In vitro*, CD8^+^ cytotoxic T cells (CTLs) separated from EBVaGC can specifically kill autologous EBV-transformed lymphoblastoid cells^4^. However, *in vivo*, adoptive cellular immunotherapy based on EBV-specific CD8^+^ CTLs has been explored with limited success^5^. Several evasion mechanisms have been proposed; one current focus in the attempt to understand this phenomenon is regulatory T cells (Tregs)^6^.

Tregs, originally called suppressor T cells, were first proposed by Gershon and Kondo in 1971, who demonstrated their ability to endow naïve animals with antigen-specific tolerance^7^. They represent a small portion of CD4^+^ T cells and constitutively express CD25 (IL-2Rα chain) on their surface^8^. The discovery of the transcription factor FOXP3, a foxhead/winged helix transcription factor, as a major marker of Treg development and function has been a significant advancement in the study of Tregs^9^. Given that FOXP3 is an intracellular molecule, the detection of Tregs requires fixation and permeabilization, hence limiting Treg isolation and *ex vivo* expansion. A recently reported cell-surface marker has resolved this issue by demonstrating that the absence or low expression of surface-expressed CD127, the α-chain of the IL-7 receptor, in combination with the high expression of CD25 can effectively distinguish Tregs from conventional CD4^+^ T cells^10^. In addition to the markers mentioned above, signatures, such as glucocorticoid-induced TNF receptor (GITR), cytotoxic T lymphocyte antigen 4 (CTLA-4) and TCR-inducible costimulatory receptor (ICOS), have gained increasing attention because of their elevated expression when Tregs are activated^11^,^12^,^13^.

In addition to their functions in the maintenance of immunological homeostasis and self tolerance, Tregs also play an important role in suppressing T cell-mediated antitumor immunity by suppressing autologous CD4^+^ helper T cells and CD8^+^ effector T cells^14^. In classical HL, the migration of Tregs towards the tumour microenvironment significantly increases in the presence of EBV^15^. This increased Treg migration is associated with the loss of EBV-specific immunity through suppression of the proliferation and IL-2 and IFN-γ secretion of EBV-specific CTLs after antigen-specific stimulation *in vitro*^16^. Further research has shown that the expression of the EBV nuclear antigen 1 (EBNA1) in HL cells mediates the upregulation of CCL20 and Treg migration through the interaction with chemokine CCL20 in HL cells and its corresponding chemokine receptor CCR6 on the Treg surface^17^. In NPC, Tregs are also increased in the tumour microenvironment and show enhanced suppressive activities against autologous CD4^+^CD25^-^ T cell proliferation^18^.

Although attention has been paid to Treg infiltration in EBV-associated HL and NPC, few studies of Tregs in EBVaGC have been reported. Haas *et al.*^19^ has reported the massive infiltration of CD8^+^T cells accompanying increased intratumoural FOXP3^+^ Treg infiltration in four cases of EBVaGC. However, the mechanism of Treg infiltration in EBVaGC has not been determined. In this study, we investigated the number of Tregs in 45 cases of EBVaGC and 45 cases of EBV-negative gastric carcinoma (EBVnGC) with matched clinicopathological parameters and found that Tregs were significantly increased in EBVaGC. We also investigated the mechanisms behind Treg accumulation within EBVaGC tumour sites and found that increased immigration and proliferation, as well as decreased emigration and apoptosis, contributed to the accumulation of Tregs in EBVaGC.

## Results

### FOXP3^+^ Tregs were increased in EBVaGC tissues

**Figure 1:**
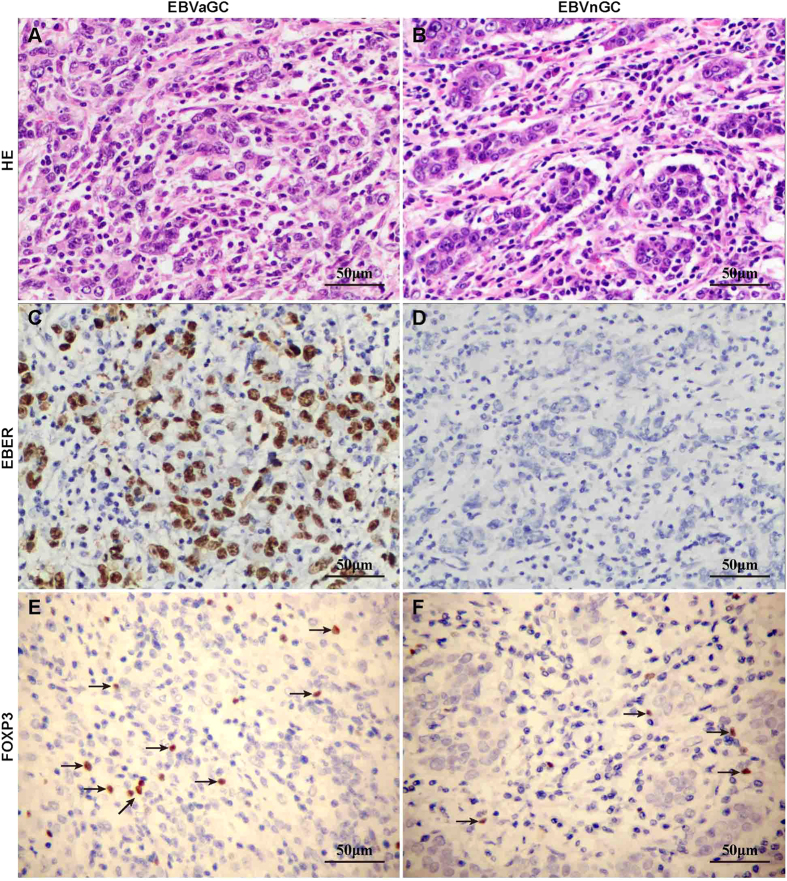
FOXP3^+^ Tregs were increased in EBVaGC tissues. Immunohistochemical staining demonstrated that the number of FOXP3^+^ Tregs in EBVaGC (left panel) is higher than that in EBVnGC (right panel). The presence of Tregs was displayed by FOXP3 staining, as indicated by arrows. (Magnification 400×)

Immunohistochemistry was used to visualize the presence of FOXP3^+^ Tregs in EBVaGC and EBVnGC. The number of FOXP3^+^ Tregs was significantly higher in EBVaGC compared with EBVnGC (15.92 ± 11.45/HPF vs. 8.45 ± 6.16/HPF, *p* = 0.001; Fig. 1). (HPF, high-power microscopic fields, 40 × 10)

### Tregs were significantly increased after co-culture with EBV (+) gastric cells

**Figure 2:**
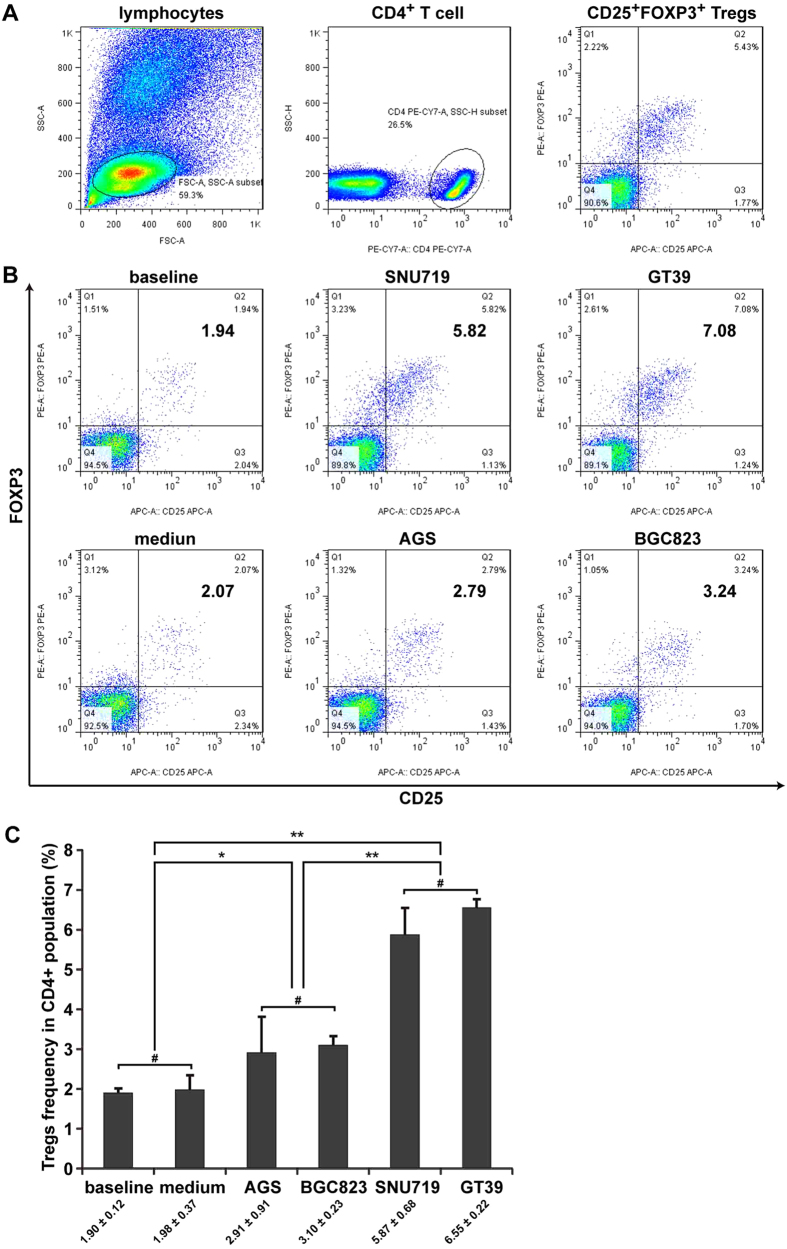
Treg levels were significantly increased after co-culture with EBV (+) gastric cells. (**A**) The gating strategy used to identify the CD4^+^CD25^+^FOXP3^+^ Tregs subset. (**B**) Representative flow cytometry dot plots in each group. (**C**) The differences in Treg frequencies among groups. Tregs in co-culture systems were significantly increased compared with baseline control (newly isolated PBMCs without further culturing) or natural control (PBMCs cultured in medium alone), whereas the frequencies of Tregs in EBV (+) SNU719 and GT39 co-culture systems were significantly higher than those in EBV (−) AGS and BGC823 co-culture systems. Error bars represent the mean ± SD, n = 3. ^#^*p* > 0.05; *p < 0.05; **p < 0.01. All *p* values were from one-way ANOVA.

In the present study, co-culture experiments with gastric cells and peripheral blood mononuclear cells (PBMCs) were performed to simulate the interaction between gastric carcinoma cells and lymphocytes *in vivo*. The frequencies of CD4^+^CD25^+^FOXP3^+^ Tregs in PBMCs after co-culture with gastric cells were evaluated by flow cytometry, and the results are expressed as the percentage of total CD4^+^ T cells. Compared to the baseline (1.90 ± 0.12%) or natural control (1.98 ± 0.37%), each co-culture system displayed an increase of Tregs in varying degrees, but the frequencies in EBV (+) co-culture systems were almost 2-fold higher than those in EBV (−) co-culture systems (SNU719 5.87 ± 0.68%, GT39 6.55 ± 0.22% vs. AGS 2.91 ± 0.91%, BGC823 3.10 ± 0.23%; Fig. 2).

### CCL22 expression was higher in EBVaGC than in EBVnGC

**Figure 3:**
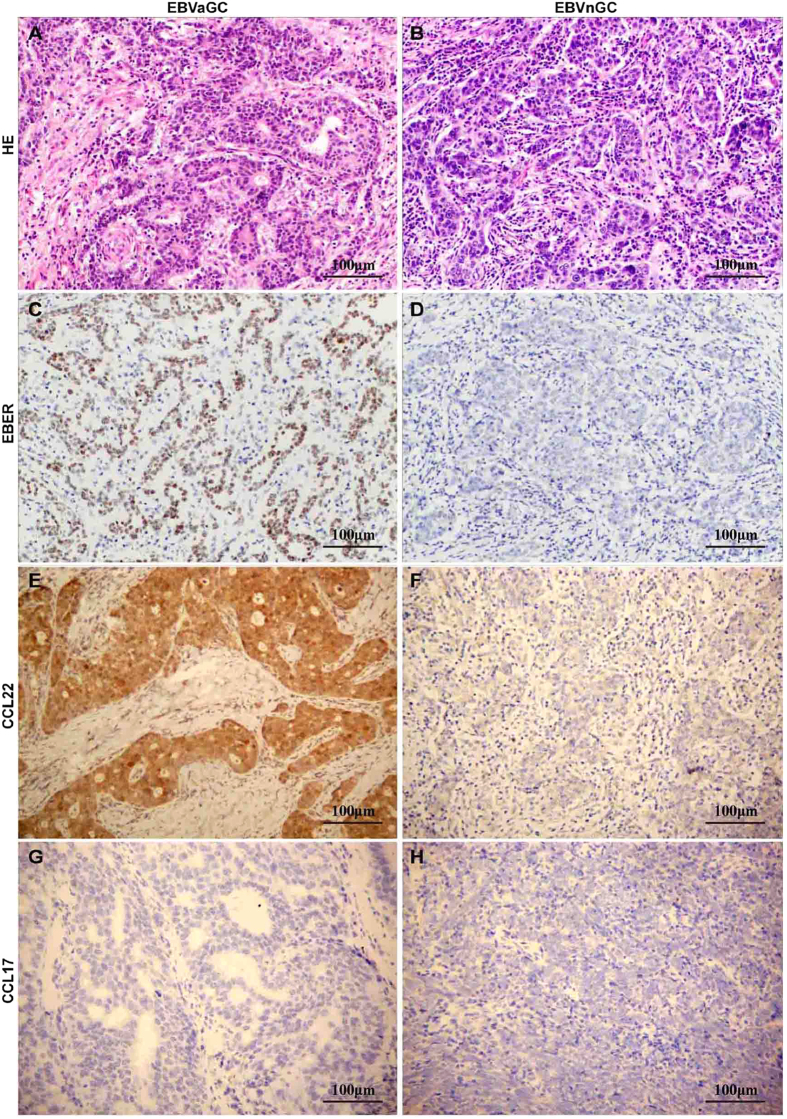
Immunohistochemical analysis of CCL22 and CCL17 expression in EBVaGC and EBVnGC tissue sections. (**A**) HE staining of a representative EBVaGC case; (**B**) HE staining of a representative EBVnGC case; (**C**) EBER *in situ* hybridization showed strong nuclear staining in almost all EBVaGC cells; (**D**) EBVnGC did not display EBER staining *in situ* hybridization; (**E**) strong staining of CCL22 in EBVaGC; (**F**) faint staining of CCL22 in EBVnGC; (**G**) absent expression of CCL17 in EBVaGC. (**H**) absent expression of CCL17 in EBVnGC. (Magnification 200×).

CCL17 and CCL22 are two major chemokines responsible for Treg migration. These molecules mediate their functionality through CCR4, a receptor upregulated in Tregs^20^. In this study, most cases of EBVaGC and EBVnGC did not display CCL17 staining, and only a few cases displayed weak to moderate staining. For CCL22, negative, weak, moderate and strong staining was found in 3, 11, 23 and 8 EBVnGC cases, respectively. The number of EBVaGC which showed negative, weak, moderate and strong staining of CCL22 was 1, 7, 20 and 17, respectively. There were more cases, with stronger staining in EBVaGC than in EBVnGC (*p* = 0.026) (Table S3, Fig. 3 & Supplementary Fig. S1).

### Enhanced CCL22 production in EBV (+) gastric cells after PBMCs stimulation associated with increased Treg migration

**Figure 4:**
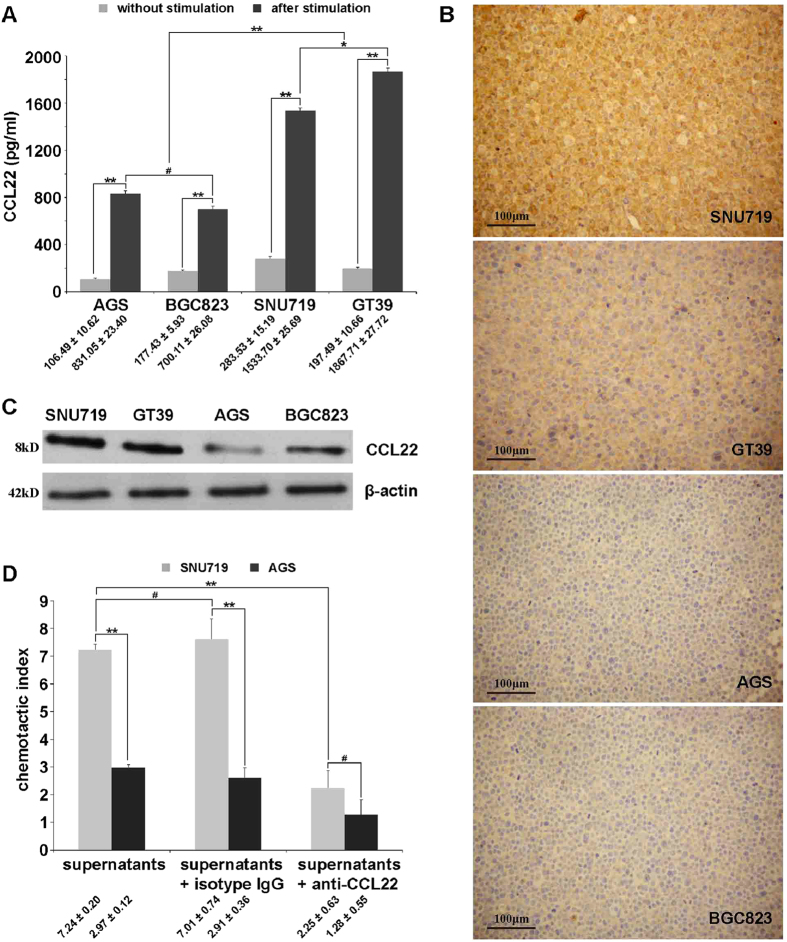
CCL22 production in gastric cells and its chemotactic effect in Treg migration. (**A**) Quantification of CCL22 levels within gastric cell supernatants with or without PBMC stimulation, assayed by ELISA. All of the gastric cells demonstrated a remarkable increase in CCL22 secretion after PBMC stimulation, especially EBV (+) SNU719 and GT39 gastric cells. (**B**) Immunohistochemical staining of CCL22 revealed stronger expression in EBV (+) SNU719 and GT39 gastric cells than in EBV (−) AGS and BGC823 gastric cells after stimulation by PBMCs. (**C**) Western blot analysis showed that the level of CCL22 protein was higher in EBV (+) SNU719 and GT39 gastric cells than that in EBV (−) AGS and BGC823 gastric cells after being stimulated by PBMCs. (**D**) After the gastric cells were stimulated by PBMCs, Treg transmigration toward EBV (+) SNU719 gastric cell supernatants was higher than that toward EBV (−) AGS gastric cell supernatants and were specifically blocked by anti-CCL22 antibody but not by isotype IgG. Error bars represent the mean ± SD, n = 3. ^#^*p* > 0.05; *p < 0.05; **p < 0.01. All *p* values were from one-way ANOVA.

In contrast to the higher expression of CCL22 in EBVaGC tissues, spontaneous CCL22 secretion of EBV (+) gastric cells without PBMC stimulation was extremely low, similar to EBV (−) gastric cells. When gastric cells were stimulated with PBMCs in advance, CCL22 production was remarkably enhanced in both EBV (+) and EBV (−) gastric cells. However, the production was 2 ~ 3-fold higher in EBV (+) groups than in EBV (−) groups (Fig. 4A). Immunohistochemical and western blot analyses of gastric cells stimulated with PBMCs also showed stronger expression of CCL22 in EBV (+) SNU719 and GT39 gastric cells than in EBV (−) AGS and BGC823 gastric cells (Fig. 4B,C).

Transwell assays of purified Tregs were used to test the chemotactic function of supernatants produced in SNU719 and AGS gastric cells previously stimulated by PBMCs. Tregs separated from PBMCs by using CD4^+^CD25^+^CD127^low/−^ MACS were identified by flow cytometry, with a purity of approximately 93.5% (Supplementary Fig. S2). As the chemotactic index in Fig. 4D shows, the supernatants derived from gastric cells pre-treated with PBMCs favoured Treg migration in both gastric cell lines, whereas there was almost 2-fold higher Treg migration in EBV (+) SNU719 than in EBV (−) AGS. The migration in these two gastric cell lines was specifically blocked by the pre-incubation of supernatants with an anti-CCL22 neutralizing mAb but not with an isotype IgG antibody.

### CD4^+^CD25^+^FOXP3^+^ Tregs underwent signature changes after co-culture with EBV (+) gastric cells

To determine whether the increased accumulation of Tregs in EBVaGC was due to impaired emigration, CD4^+^ CD25^+^ FOXP3^+^ Tregs from the co-culture systems were analysed for the expression of the typical lymphocyte homing receptors CCR7 and CD62L. Both these receptors were downregulated in Tregs after co-culture compared with PBMCs cultured in medium alone. However, there was a greater decrease in CCR7 expression in Tregs co-cultured with EBV (+) SNU719 and GT39 gastric cells than those co-cultured with EBV (−) AGS and BGC823 gastric cells, whereas no difference in CD62L expression was observed among the co-culture systems, regardless of EBV infection status.

The level of the functional associated molecules CTLA-4 and GITR was also determined. Compared to PBMCs cultured in medium alone, there was enhanced expression of CTLA-4 and GITR in Tregs during co-culture with both EBV (+) and EBV (−) gastric cells, but the expression was higher in EBV (+) co-culture systems than in EBV (−) co-culture systems.

**Figure 5:**
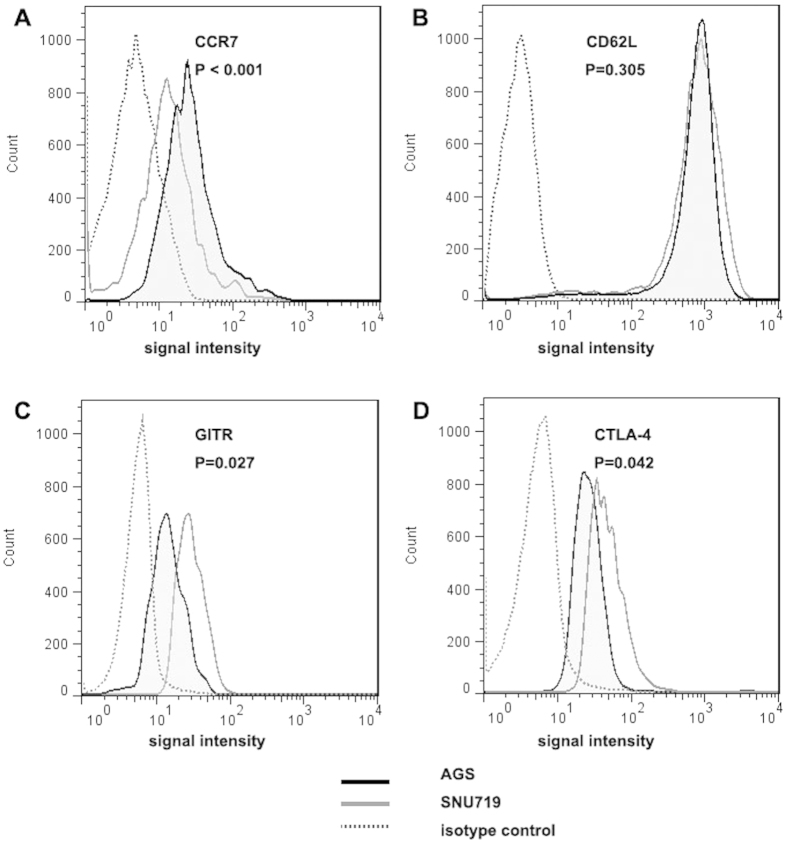
Expression of lymphocyte homing receptors CCR7 and CD62L and GITR and CTLA-4 signatures on Tregs. Representative flow cytometry histograms from EBV (+) SNU719 gastric cells and EBV (−) AGS gastric cells were shown. (**A**) Expression of CCR7 on the Treg surface was down-regulated after co-culture with EBV (+) SNU719 gastric cells compared with co-culture with EBV (−) AGS gastric cells. (**B**) No difference was observed on CD62L expression in EBV (+) SNU719 and EBV (−) AGS co-culture systems. (**C,D**) Expression of GITR and CTLA-4 were far more upregulated after co-culture with EBV (+) SNU719 gastric cells than with EBV (−) AGS gastric cells.

The expression of these four signatures is summarized in Table 1. Representative flow cytometry histograms from EBV (+) SNU719 gastric cells and EBV (−) AGS gastric cells demonstrating these four signatures expression are shown in Fig. 5.

### Treg proliferation was enhanced after EBV (+) gastric cell supernatant stimulation

**Figure 6:**
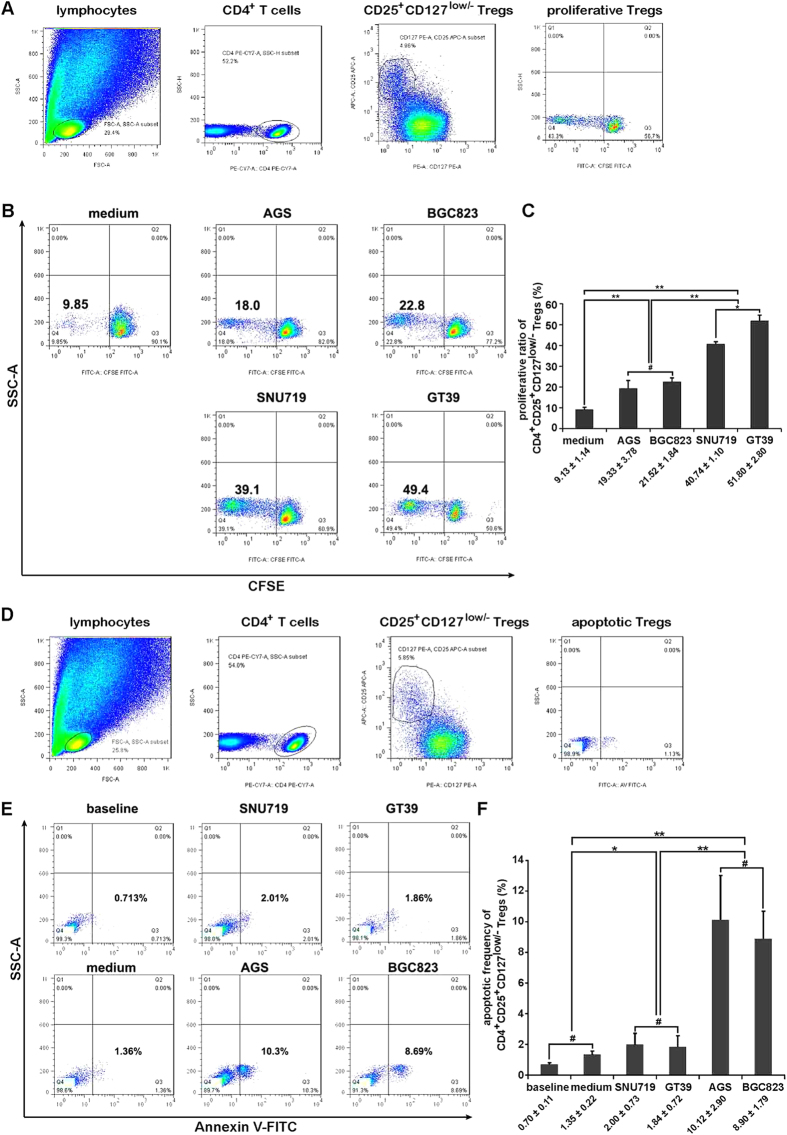
Proliferation and apoptosis of Tregs after co-culture experiments. (**A**) Gating strategy for CD4^+^CD25^+^CD127^low/−^ Treg identification as well as CFSE cell proliferation analysis in a Treg subset. (**B**) Representative flow cytometry dot plots showing the proliferation of Tregs in each culture system. (**C**) Histogram showing that EBV (+) SNU719 and GT39 gastric cell supernatants were more effective in promoting Treg proliferation than EBV (−) AGS and BGC823 gastric cell supernatants. (**D**) Gating strategy for CD4^+^CD25^+^CD127^low/−^ Treg identification as well as Annexin V binding analysis in a Treg subset. (**E**) Representative flow cytometry dot plots of apoptotic Tregs under different culture conditions. (**F**) Differences in apoptotic Treg frequencies among different treatments. The apoptotic Treg frequency increased in co-culture systems compared with baseline (newly isolated PBMCs without further culture) and natural (PBMCs cultured in medium alone) controls, and it was significantly higher in EBV (−) AGS and BGC823 gastric cells than that in EBV (+) SNU719 and GT39 gastric cells. Error bars represent the mean ± SD, n = 3. ^#^*p* > 0.05; *p < 0.05; **p < 0.01. All *p* values were from one-way ANOVA.

Compared to that of PBMCs cultured in medium alone (9.13 ± 1.14%), the proliferation index in the co-culture systems was significantly increased. However, it was notably higher in EBV (+) SNU719 and GT39 cell supernatants (40.74 ± 1.10% and 51.80 ± 2.80%, respectively) compared to EBV (−) AGS and BGC823 cell supernatants (19.33 ± 3.78% and 21.52 ± 1.84%, respectively) (Fig. 6A–C).

### Tregs in EBV (+) co-culture systems were more resistant to apoptosis

We examined Annexin V binding to CD4^+^CD25^+^CD127^low/−^ Tregs in PBMCs, which were identified as apoptotic Tregs. There was an increase in the Treg apoptotic frequency after co-culture with both EBV (+) and EBV (−) gastric cells when compared to the baseline or natural control. However, Tregs in EBV (+) co-culture systems were more resistant to apoptosis than EBV (−) co-culture systems (Fig. 6D–F).

## Discussion

In the present study, we showed that Tregs were significantly increased in EBVaGC compared to EBVnGC. In NPC, another EBV-associated epithelial malignancy, Tregs are also increased^18^. In classical HL, the migration of Tregs towards the tumour microenvironment is significantly increased in the presence of EBV^15^. These findings suggest that Treg accumulation in tumour sites might be a common phenomenon in EBV-associated malignancies. However, the mechanism of the accumulation of infiltrating Tregs in EBV-associated malignancies is still poorly understood.

In this study, EBV (+) gastric cells were used as *ex vivo* models for EBVaGC, and co-culture experiments with PBMCs and gastric cells were performed. We found that increased recruitment could be attributed to higher CCL22 production by EBVaGC cells, decreased emigration caused by downregulated lymphocyte homing receptor CCR7 on the Treg surface, higher Treg proliferation rates and lower apoptosis rates at tumour sites.

CCL17 and CCL22 are two critical chemokines that modulate the migration of Tregs through their corresponding receptor CCR4 on the Treg surface^21^. In this study, CCL22 but not CCL17 showed higher expression in EBVaGC than in EBVnGC. In addition, the CCL22 production in EBV (+) gastric cells was increased and was significantly higher than that in EBV (−) gastric cells after co-culture with PBMCs. Moreover, as demonstrated by transwell assays, the enhanced CCL22 production in EBV (+) gastric cells after PBMC stimulation caused increased Treg migration *in vitro*. These findings suggested that the higher expression of CCL22 in EBVaGC cells might be responsible for the increased migration of Tregs in EBVaGC. It has been reported that EBV latent membrane protein 1 (LMP1), which is functionally similar to CD40, can act as a constitutively activated receptor to activate NF-κB signalling and upregulate downstream genes, including CCL22^22^,^23^. However, LMP1 was not expressed in EBVaGC. Therefore, the mechanisms of enhanced CCL22 production in EBVaGC need to be further investigated.

As a subset of lymphocytes, Tregs constitutively express the lymphocyte homing receptors CCR7 and CD62L, which enable Tregs to continuously recirculate through the lymph nodes, where they can encounter antigens and become activated^24^. Down-regulation of these trafficking receptors causes Treg accumulation in tumour sites instead of peripheral circulation in various tumours, including NPC^18^, colorectal cancer^25^ and squamous cell carcinoma of the neck^26^. In our experiments, a significant decrease of CCR7 in Tregs was detected in EBV (+) co-culture systems compared to EBV (−) co-culture systems, whereas no difference of CD62L expression existed between EBV (+) and EBV (−) groups. Therefore, we propose that the increase in Tregs in EBVaGC may be due to the decreased expression of CCR7 in Tregs. It has been reported that lymphocyte activation is associated with CCR7 down-regulation in squamous cell carcinoma of the head and neck, causing the functional cells to reside in tumour sites and exert immune functions^27^. Our study has also revealed that functional associated molecules CTLA-4 and GITR, whose expression was elevated when Tregs were activated, were higher in EBV (+) co-culture systems than in EBV (−) co-culture systems. This finding suggests that Treg activation is associated with CCR7 down-regulation in EBVaGC, which might cause Tregs to reside in EBVaGC tumour sites and exert immune functions.

In comparison to effector T cells, Tregs are difficult to expand due to their unique characteristic of anergy^28^. However, the proliferation analysis in this study showed that the proliferative Tregs significantly increased after stimulation with EBV (+) gastric cell supernatants compared to EBV (−) gastric cell supernatants, which is another possible reason for the accumulation of Tregs in EBVaGC. It has been reported that immunoinhibitory cytokines, such as IL-10 and TGF-β, not only play important roles in Treg suppressive function but also are critical in Treg generation and expansion^29^. We showed that the production of IL-10 and TGF-β significantly increased in EBV (+) co-culture systems compared to EBV (−) co-culture systems (Supplementary Fig. S3). These increased cytokine levels provide important support for Treg proliferation.

Furthermore, the apoptosis of Tregs in the EBV (+) co-culture systems was much lower than that in EBV (−) co-culture systems. This finding may provide an additional explanation for the increase of Tregs in EBVaGC. Nocentini *et al.*^30^ have reported that GITR is a member of the tumour necrosis factor/nerve growth factor receptor family involved in the regulation of T cell receptor-mediated cell death. The constitutive expression of a transfected GITR gene induces resistance to anti-CD3 mAb-induced apoptosis. Ronchetti *et al.*^31^ have found that activated GITR^(−/−)^ T lymphocytes are more sensitive to activation-induced cell death than GITR^(+/+)^ T lymphocytes. Our data showed that GITR expression was much higher in EBV (+) co-culture systems, which may provide an additional explanation for the decreased apoptosis of Tregs within EBVaGC.

In conclusion, the present study reports an increased infiltration of Tregs in EBVaGC compared with EBVnGC. Increased immigration and proliferation, as well as decreased emigration and apoptosis, might contribute to the accumulation of Tregs in EBVaGC. Because Tregs have been regarded as a critical hurdle in CD8^+^ CTL-mediated anti-tumour immunity, a better understanding of their accumulation would favour strategies designed to regulate the balance between Tregs and effector T cells within EBVaGC, thus promoting the future immunotherapy in EBVaGC.

## Materials and Methods

### Ethics statement

The use of human subjects was approved by the Clinical Research Ethics Committee of the Third Affiliated Hospital, Sun Yat-sen University, Guangzhou, China. Written informed consent was obtained from all the patients and healthy volunteers, and the ethical guidelines under the Declaration of Helsinki were followed.

### Tissue specimens

Formalin-fixed, paraffin-embedded gastric adenocarcinoma specimens collected in the Second and Third Affiliated Hospitals of Sun Yat-sen University and the Guangzhou First Municipal People’s Hospital, Guangzhou, China, were used to determine the EBV infection status by *in situ* hybridization assay for EBER-1. EBER-1 (+) and EBER-1 (−) cases were defined as EBVaGC and EBVnGC, respectively. Of the 676 cases, 45 cases (6.7%) were identified as EBVaGC^32^. In the present study, 45 cases of EBVaGC together with 45 cases of EBVnGC with matched clinicopathological parameters were selected for immunohistochemistry investigation. The clinicopathological characteristics of EBVaGC and EBVnGC are presented in Table S1.

### Immunohistochemical staining and scoring

Immunohistochemical analysis was performed using an Envision system (Dako Envision) in accordance with the manufacturer’s instructions. 3,3-diaminobenzidine (DAB) was used as a chromogen. The primary antibodies used in the present study and their retrieval methods, as well as their dilutions, are shown in Table S2. PBS was used instead of the primary antibody as the negative control. Formalin-fixed, paraffin-embedded sections from normal human tonsil tissue were used as positive controls.

FOXP3 expression was located in the nuclei of TILs. FOXP3-positive lymphocytes (Tregs) in 10 randomly selected high-power microscopic fields (HPFs, 40×10) were counted, and the mean number of positively stained lymphocytes per HPF was calculated^33^.

CCL17 and CCL22 were both expressed in the cytoplasm and/or the nuclei. The semi-quantitative scores method was used to evaluate the expression of CCL17 and CCL22 based on the percentage and staining intensity of positive tumour cells. The percentage of positive tumour cells was graded as follows: 0, none; 1, 1 ~ 24%; 2, 25 ~ 49%; 3, 50 ~ 74%; and 4, 75 ~ 100%. The staining intensity was scored as follows: 0, absent; 1, weak; 2, moderate; and 3, strong. The raw data were converted to a total immunoreactive score by multiplying the score of the percentage of positive cells and the score of the staining intensity, as previously reported^34^. The total immunoreactive score, which ranged from 0 to 12, was divided into four categories as follows: -, 0; +, 1 ~ 4; ++, 5 ~ 8; +++, 9 ~ 12.

### Collection of PBMCs

Human peripheral blood buffy coat samples from healthy volunteers were obtained from Guangzhou Blood Centre for PBMCs isolation. The PBMCs were isolated by Ficoll-Hypaque (MP Biomedical) gradient separation solution, retrieved from the interface and washed twice in heparinized PBS without calcium and magnesium. They were then counted to evaluate their viability in the presence of trypan blue dye and were processed either for immediate use or cryopreserved for further experiments.

### Cell lines

The EBV (+) human gastric carcinoma cell lines SNU719 and GT39, EBV (−) human gastric carcinoma cell lines AGS and BGC823, were used in this study. Both SNU-719 and GT39 are naturally derived EBV-infected cell lines, with the EBV genome latently infected as an episome. SNU719 was obtained from a patient with primary gastric carcinoma. GT39 was also derived from gastric tissues with carcinoma, although it was derived from noncancerous portions of gastric carcinoma tissues. AGS and BGC823 were established from the tissue of patients with primary gastric carcinoma without EBV infection. All of the cell lines are adherent.

### Cell culture

Cell lines SNU719, GT39, AGS and BGC823, as well as PBMCs, were cultured in RPMI-1640 medium (Invitrogen) supplemented with 10% FBS (Invitrogen) at 37 °C in a 5% CO_2_ incubator. The gastric carcinoma cell lines used in the following studies are referred to as “gastric cells”.

### Co-culture

To simulate the *in vivo* microenvironment and investigate the interaction between infiltrated lymphocytes and tumour cells, 2 × 10^5^ gastric cells per well in 2 ml medium were added to 12-well plates, and a 10-fold excess of PBMCs in 1 ml of medium was added after gastric cell adherence. After co-culture for 72 h, PBMCs in the supernatant were retrieved, and the frequencies, phenotypes and apoptosis of Tregs in PBMCs were analysed by flow cytometry. Newly isolated PBMCs without further culture and PBMCs cultured in medium alone were used as the baseline and natural controls, respectively.

### Extracellular and intracellular detection of CCL22 production in gastric cells

For the detection of CCL22 production, gastric cells after PBMCs simulation were prepared as follows: after co-culture with a 10-fold excess of PBMCs for 24 h, gastric cells were retrieved by thoroughly washing off the PBMCs, and 2×10^5^ gastric cells per well in 1 ml of medium were re-incubated in 12-well plates and cultured for another 48 h. For gastric cells without PBMC stimulation, 2 × 10^5^ cells per well in 1 ml of medium were incubated for 48 h in 12-well plates without previous stimulation by PBMCs.

For the extracellular detection of CCL22 production, supernatants from gastric cells with or without PBMC simulation were collected by centrifuging at 300×g for 10 min and filtered with a 0.22 μm filter. The CCL22 levels were quantified using Quantikine Immunoassay kits (R&D Systems) according to the manufacturer’s instructions. The remainder of the supernatants from SNU719 and AGS cells stimulated by PBMCs was used for the transwell analysis.

For the intracellular detection of CCL22, both immunohistochemical staining and western blot analysis were conducted. For the immunohistochemical staining, PBMC-stimulated gastric cells were collected, washed in PBS 3 times, fixed in 10% neutral formalin for 30 min, and then centrifuged at 300 × g for 10 min. The precipitate was carefully removed from the bottom of the tube, wrapped in tissue paper and placed in a labelled plastic cassette for paraffin processing as for a routine biopsy specimen. A 4 μm-thick section was cut from each cell block and used for immunohistochemistry. For western blot analysis, whole-cell proteins were harvested by homogenizing cells in lysis buffer. Protein concentrations were determined using the Bio-Rad protein assay (Bio-Rad, Richmond, CA, USA). Equal amounts of samples were separated on 8% SDS-PAGE gels. The proteins were then transferred to polyvinylidene fluoride membranes (Millipore, Bedford, MA, USA) and probed with rabbit polyclonal anti-CCL22 (1:1000; Abcam, UK) and rabbit monoclonal anti-β-actin (1:1000; Cell Signaling Technology, Beverly, MA, USA). Finally, the blots were probed with HRP-conjugated secondary antibodies and visualized using ECL (Thermo).

### Transwell assay

Transwell assays were performed on EBV (+) SNU719 and EBV (−) AGS cell supernatants with PBMCs stimulation. Tregs were separated from PBMCs by using CD4^+^CD25^+^CD127^low/−^ Magnetic Beads Separation System (MACS) (Miltenyi Biotec). Detailed separation procedures were as follows. First, the PBMCs were treated with a cocktail of biotinylated antibodies and Anti-Biotin MicroBeads to deplete non-CD4^+^ and CD127^high^ cells. Second, the flow-through fraction of pre-enriched CD4^+^CD127^low/−^ T cells was labelled with CD25 MicroBeads for subsequent positive selection of CD4^+^CD25^+^CD127^low/−^ regulatory T cells. The purity of Tregs was determined by flow cytometry. Twelve-well chemotaxis chambers with 0.4-μm porous membranes (Corning Costar) were used to measure the migration of purified Tregs toward gastric cell supernatants. Briefly, the lower chambers of plates were filled with 500 μl of conditioned supernatants or medium alone, then 5 × 10^5^ Tregs in 100 μl medium were added to the upper chambers and left to migrate at 37 °C for 6 h. The number of migrated cells in the lower chamber was counted under a microscope, and the chemotactic index was expressed as the ratio of cells that migrated in the presence of conditioned supernatants compared to those that migrated in the presence of medium alone. In blockade experiments, 5 μg/ml anti-CCL22 (Abcam) or 5 μg/ml of its isotype control IgG (Dako) was incubated for 30 min with conditioned supernatants before Tregs were added in the upper chambers.

### Flow cytometry analysis

PBMCs were stained with the following anti-human monoclonal antibodies to determine the frequencies and the phenotypes of Tregs by flow cytometry: anti-CD4-PE-Cy7, anti-CD25-APC, anti-CD127-PE, anti-CD197 (CCR7)-PE and anti-CD62L-PE were used for extracellular staining; anti-FOXP3-Alexa Fluor 488, anti-CD152 (CTLA-4)-PE and anti-CD357 (GITR)-PE were used for intra-cellular staining. Briefly, different treatments of PBMCs were recovered and resuspended in 100 μl buffer solution, then incubated with antibodies for 30 min at 4 °C for extracellular surface staining; for intracellular staining, PBMCs were first incubated with antibodies against surface markers, and after extensive washing, cells were fixed and permeabilized with an intracellular staining buffer set kit following the instruction manuals. The antibodies used for intracellular staining were added to the cells and incubated for another 30 min. All the antibodies and the buffer were purchased from eBioscience.

Detection was performed on a flow cytometer (BD-LSR II), and the analysis was performed by Flowjo software version 7.6 (Tree Star). To identify the Tregs, a lymphocyte gate was set by characteristic forward and side scatter followed by gating on CD4^+^ T cells. The cells were then analysed for the co-expression of CD25^+^FOXP3^+^ or CD25^+^CD127^low/−^. The expression of signatures, including CCR7, CD62 L, GITR and CTLA-4, were analysed on the basis of CD25^+^FOXP3^+^ T cell subset. Considering the fluorescence quenching effects of fixation and permeabilization during FOXP3 staining, the surface marker CD127 was used for Treg labelling instead of FOXP3; therefore, the proliferation and apoptosis analyses were based on the CD25^+^CD127^low/−^ T cell subset.

### Proliferation assay

The CFSE Cell Proliferation Assay and Tracking Kit (KeyGEN Biotech) was used for the detection of Treg proliferation. Briefly, 5 × 10^6^ PBMCs per well stained with CFSE in 2 ml of medium were seeded in 6-well plates; then, 2 ml of supernatant derived from EBV (+) SNU719 or GT39 or EBV (−) AGS or BGC823 gastric cells was added to stimulate proliferation. After culturing for 7 days, PBMCs were collected for staining by CD4-PE-Cy7, CD25-APC, and CD127-PE antibodies, and the proliferation of Tregs was assessed by flow cytometry. The percentage of CD4^+^CD25^+^CD127^low/−^ Tregs with lower CFSE staining compared to the total CD4^+^CD25^+^CD127^low/−^ Tregs was defined as the proliferation index.

The gastric cell supernatants used above were prepared as follows: 2 × 10^5^ gastric cells per well in 1 ml of medium were added to 12-well plates. After a 24 h incubation, supernatants were retrieved and filtered with a 0.22 μm filter. The supernatants were then added to PBMCs to stimulate proliferation. PBMCs cultured in medium alone were also tested for their natural proliferation without any stimulation.

### Apoptosis assay

Apoptosis of Tregs in the PBMCs co-cultured with gastric cells was determined using the Annexin V-FITC Apoptosis Detection Kit (KeyGEN Biotech). Briefly, after 72 h co-culture with gastric cells, PBMCs were isolated from the 12-well plates and stained with CD4-PE-Cy7, CD25-APC and CD127-PE antibodies for Treg labelling, together with Annexin V-FITC for the apoptotic analysis. The detection was performed by flow cytometry. Apoptotic Tregs were also detected in newly isolated PBMCs and PBMCs cultured in medium for 72 h alone, which represented the baseline and natural controls, respectively.

### Statistical analysis

Data are expressed as the mean ± SD. Comparisons of the number of Tregs between EBVaGC and EBVnGC tissue sections were made by Student’s *t* test. Differences in CCL17 or CCL22 expression between EBVaGC and EBVnGC tissue sections were analysed using the rank sum test. One-way ANOVA was used for multiple comparisons among several gastric cell lines. The results from each cell line were the mean of the assays in triplicate. The results were considered to be statistically significant at a *p* value of less than 0.05. All analyses were performed using SPSS 13.0 software (SPSS, Inc., Chicago, IL).

## Additional Information

**How to cite this article**: Zhang, N.-na *et al.* Accumulation Mechanisms of CD4^+^CD25^+^FOXP3^+^ Regulatory T Cells in EBV-associated Gastric Carcinoma. *Sci. Rep.* **5**, 18057; doi: 10.1038/srep18057 (2015).

## Supplementary Material

Supplementary Information

## Figures and Tables

**Table 1:** Signatures of Tregs in PBMCs co-cultured with EBV (+) or EBV (−) gastric cells or cultured in medium alone (%) (Mean ± SD).

Group	EBV (+)		EBV (−)		medium
	SNU719	GT39	AGS	BGC823	
CCR7*	23.73 ± 1.84	28.33 ± 3.71	54.00 ± 6.40	42.13 ± 1.69	85.28 ± 2.89
CD62L†	94.20 ± 1.90	92.33 ± 1.39	93.53 ± 1.03	95.83 ± 0.09	97.10 ± 0.73
GITR‡	85.30 ± 0.40	84.90 ± 0.27	54.40 ± 7.27	66.75 ± 8.50	39.30 ± 0.60
CTLA-4§	89.55 ± 0.85	89.13 ± 2.62	34.30 ± 4.06	39.23 ± 1.13	29.90 ± 2.32

^*^p values from one-way ANOVA. SNU719 vs. GT39: *p* > 0.05; AGS vs. BGC823: *p* < 0.05; EBV (+) vs. EBV (−): *p* < 0.01; EBV (+) vs. medium: *p* < 0.01; EBV (−) vs. medium: *p* < 0.01.

^†^*p values* from one-way ANOVA. SNU719 vs. GT39: *p*>0.05; AGS vs. BGC823: *p*>0.05; EBV (+) vs. EBV (−): *p* > 0.05; EBV (+) vs. medium: *p* < 0.05; EBV (−) vs. medium: *p* < 0.05.

^‡^*p values* from one-way ANOVA. SNU719 vs. GT39: *p*>0.05; AGS vs. BGC823: *p*>0.05; EBV (+) vs. EBV (−): *p* < 0.01; EBV (+) vs. medium: *p* < 0.01; EBV (−) vs. medium: *p* < 0.01.

^§^*p values* from one-way ANOVA. SNU719 vs. GT39: *p*>0.05; AGS vs. BGC823: *p* > 0.05; EBV (+) vs. EBV (−): *p* < 0.01; EBV (+) vs. medium: *p* < 0.01; EBV (−) vs. medium: *p* < 0.01.
