# Supplementary material for: Accumulation Mechanisms of CD4+CD25+FOXP3+ Regulatory T Cells in EBV-associated Gastric Carcinoma
Source: Sci Rep. 2015 Dec 17;5:18057. doi: 10.1038/srep18057 (PMC4682180; doi:10.1038/srep18057)
Supplement: Supplementary Information [file srep18057-s1.doc]

**Original article**

**Title of the paper**

Accumulation Mechanisms of CD4^+^CD25^+^FOXP3^+^ Regulatory T Cells in EBV-associated Gastric Carcinoma

**Authors’ names and institutional affiliations**

Na-na Zhang^1^, Jian-ning Chen^1^, Lin Xiao^1,2^, Fang Tang^1,3^, Zhi-gang Zhang^1^, Yi-wang Zhang^1^, Zhi-ying Feng^1^, Ye Jiang^1^ , Chun-kui Shao^1*^

^1^Department of Pathology, The Third Affiliated Hospital, Sun Yat-sen University, No. 600 Tianhe Road, Guangzhou 510630, China

^2^Present address: Henan Key Laboratory of Tumor Pathology, Department of Pathology, The First Affiliated Hospital, Zhengzhou University, No. 1 Jianshedong Road, Zhengzhou 450052, China

^3^Present address: Department of Pathology, Affiliated Hospital of Guilin Medical University, No.15 Lequn Road, Guilin 541001, China

^*^Corresponding author

**The email address of corresponding author**

Chun-kui Shao：[chunkuishao2011@163.com](mailto:chunkuishao2011@163.com)

**Supplementary Figures**

**FIGURE S1. Representative immunohistochemical images for different staining of CCL22 and CCL17 in EBVaGC tissue sections.** Representative images for weak (*A*), moderate (*B*) and strong (*C*) staining of CCL22, as well as representative images for weak (*D*) and moderate (*E*) stainings of CCL17 were shown. (Magnification 200×)


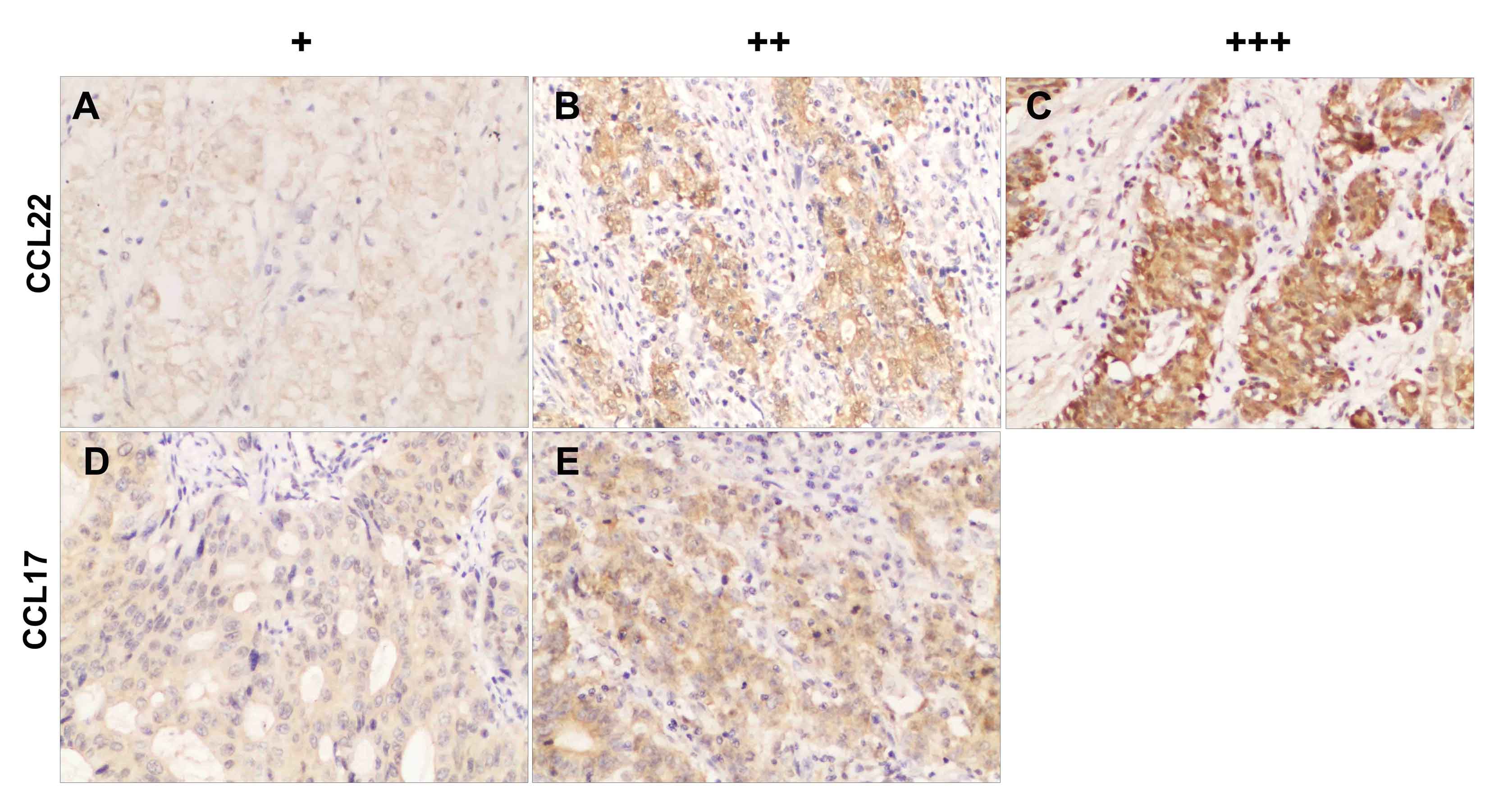


**FIGURE S2. Flow cytometry identification of purified Tregs sorted by MACS.** Tregs separated from PBMCs by using CD4^+^CD25^+^CD127^low/-^ MACS were identified by flow cytometry, with a purity of approximately 93.5%.


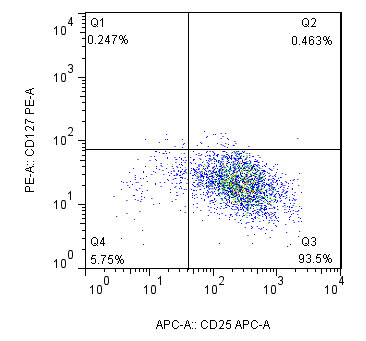


**FIGURE S3.** **IL-10 and TGF-β productions were significantly increased in EBV (+) co-culture systems.** Quantification of IL-10 and TGF-β secreted by gastric cells or PBMC cultured alone as well as in co-culture systems was performed by ELISA assays. Both the gastric cells and PBMC had the ability to secret IL-10 (*A*) and TGF-β (*B*) in low levels, while the cytokines secretion were significantly increased in the co-culture systems, especially in EBV (+) co-culture systems. Error bars represent mean ± SD. *, *p* < 0.05; **, *p* < 0.01.


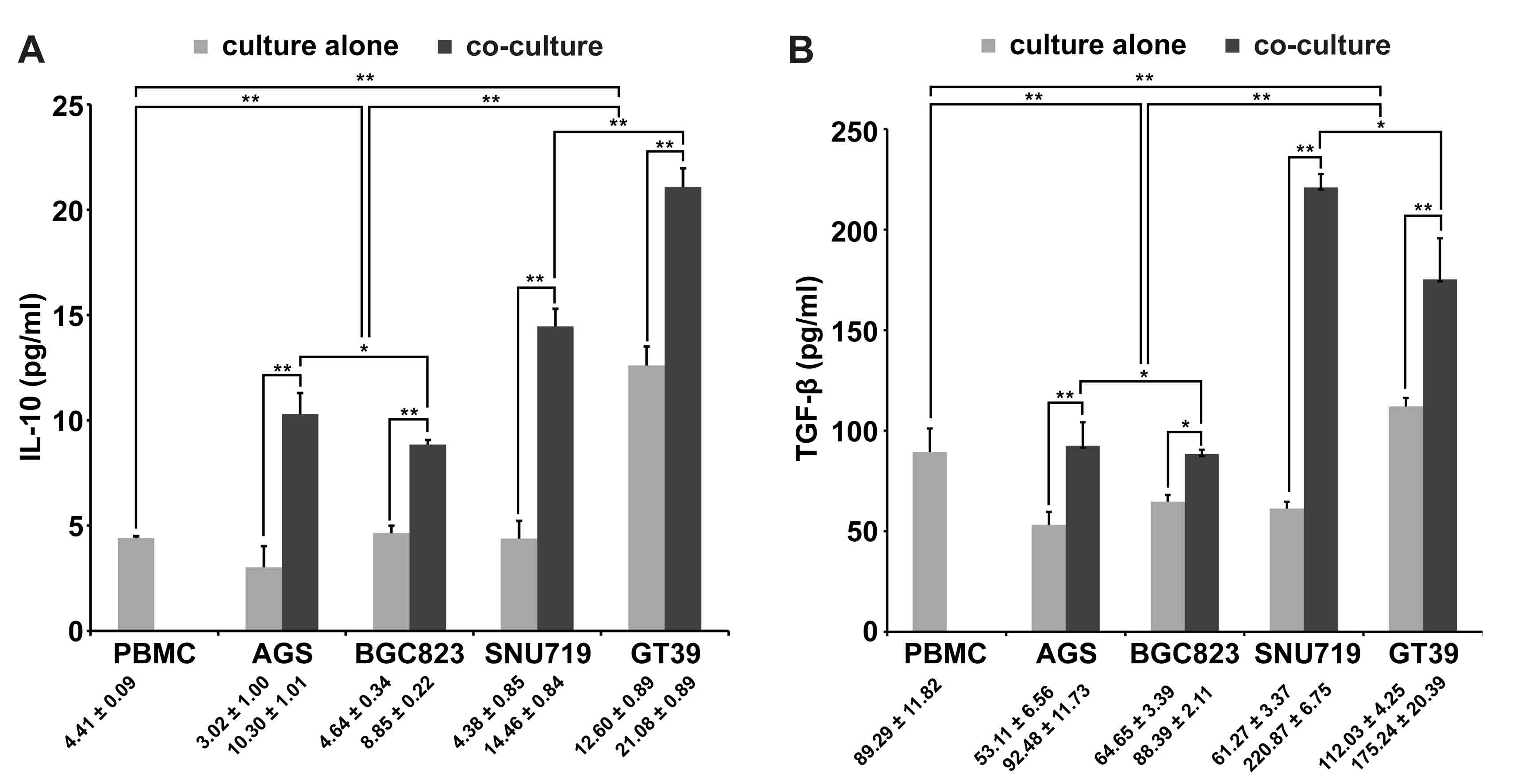


**Supplementary Tables**

**Table S1. *Clinicopathological characteristics of EBVaGC and EBVnGC***

| **Variables** | **Total** | **EBVaGC**  **(n=45)** | **EBVnGC**  **(n=45)** | ***p*^*^** |
| --- | --- | --- | --- | --- |
| Gender |  |  |  |  |
| Female | 21 | 8 (17.8%) | 13 (28.9%) | 0.213 |
| Male | 69 | 37 (82.2%) | 32 (71.1%) |  |
| Age (years) |  |  |  |  |
| ≤40 | 24 | 11 (24.4%) | 13 (28.9%) | 0.887 |
| 40～60 | 47 | 24 (53.3%) | 23 (51.1%) |  |
| >60 | 19 | 10 (22.2%) | 9 (20%) |  |
| Location |  |  |  |  |
| Cardia | 28 | 14 (31.1%) | 14 (31.1%) | 0.690 |
| Body | 23 | 14 (31.1%) | 9 (20%) |  |
| Antrum | 34 | 15 (33.3%) | 19 (42.2%) |  |
| Whole^†^ | 5 | 2 (4.4%) | 3 (6.7%) |  |
| Histology |  |  |  |  |
| Intestinal | 22 | 8 (17.8%) | 14 (31.1%) | 0.116 |
| Diffuse | 68 | 37 (82.2%) | 31 (68.9%) |  |
| Stage (pTNM) |  |  |  |  |
| I | 5 | 1 (2.2%) | 4 (8.9%) | 0.257 |
| II | 21 | 8 (17.8%) | 13 (28.9%) |  |
| III | 43 | 23 (51.1%) | 20 (44.4%) |  |
| IV | 21 | 13 (28.9%) | 8 (17.8%) |  |

**p*-values were obtained from chi-square tests.

^†^Cases involved the whole stomach.

***Table S2. Antibodies used for immunohistochemical study***

| **Antibody** | **Retrieval methods*** | **Dilution** | **Species** | **Clone No.** | **Source** | **Positive expression** |
| --- | --- | --- | --- | --- | --- | --- |
| **FOXP3** | HP EDTA | 1:100 | mouse | 206D | Santa Cruz, USA | Nucleus |
| **CCL22** | HP EDTA | 1:50 | rabbit | polyclonal | Abcam, UK | Cytoplasm and/or nucleus |
| **CCL17** | HP EDTA | 1:50 | goat | polyclonal | Santa Cruz, USA | Cytoplasm and/or nucleus |

*HP EDTA: Retrieve in Tris/EDTA antigen retrieval solution (pH 8.0) under high pressure for 5 min.

**Table S3. *The expression of CCL17 and CCL22 in EBVaGC and EBVnGC***

| **Group** | **n** | **CCL17*** | | | |  | **CCL22**^†^ | | | |
| --- | --- | --- | --- | --- | --- | --- | --- | --- | --- | --- |
|  |  | - | + | ++ | +++ |  | - | + | ++ | +++ |
| **EBVaGC** | 45 | 39 | 5 | 1 | 0 |  | 1 | 7 | 20 | 17 |
| **EBVnGC** | 45 | 38 | 4 | 3 | 0 |  | 3 | 11 | 23 | 8 |

*rank sum test, *p*=0.523

^†^rank sum test, *p*=0.026
